# *Gliomap-GAN*: A conditional generative adversarial network to visualize glioblastoma’s cell density from contrast-enhanced magnetic resonance imaging

**DOI:** 10.1093/noajnl/vdaf227

**Published:** 2025-10-21

**Authors:** Manabu Kinoshita, Keisuke Miyake, Wataru Ide, Hideyuki Arita, Kayako Isohashi, Jun Hatazawa, Haruhiko Kishima

**Affiliations:** Department of Neurosurgery, Asahikawa Medical University, Asahikawa, Japan; Department of Neurosurgery, Kagawa University Faculty of Medicine/Graduate School of Medicine, Kita District, Japan; Department of Neurosurgery, Hokuto Hospital, Obihiro, Japan; Department of Neurosurgery, Osaka International Cancer Institute, Osaka, Japan; Department of Radiology, Osaka University, Graduate School of Medicine, Suita, Japan; Department of Physics, Osaka University, Graduate School of Science, Suita, Japan; Department of Neurosurgery, Osaka University, Graduate School of Medicine, Suita, Japan

**Keywords:** ^11^C-methionine positron emission tomography, conditional generative adversarial network, glioblastoma, magnetic resonance imaging, non-contrast enhancing tumor

## Abstract

**Background:**

^11^C-methionine positron emission tomography is one of the most reliable imaging modalities for ­glioblastoma visualization. This investigation aimed to generate an ^11^C-methionine positron emission tomography-like image, “*Gliomap*,” from contrast-enhanced magnetic resonance imaging via a conditional Generative Adversarial Network (*Gliomap-GAN*).

**Methods:**

Eighty-one newly diagnosed glioblastoma patients with preoperative contrast-enhanced magnetic resonance imaging and ^11^C-methionine positron emission tomography were retrospectively collected. T1-weighted, T2-weighted, and Gd-enhanced T1-weighted images were co-registered and intensity normalized, followed by the creation of a contrast-enhancement subtraction map. They were used as source data to train *Gliomap-GAN,* targeting the corresponding ^11^C-methionine positron emission tomography image. The training dataset comprised 2459 images augmented to 4918 pairs by mirroring. The test dataset consisted of 593 pairs. Furthermore, an additional five patients with 16 image-guided sampled tissues were used for histological validation of the generated *Gliomap*.

**Results:**

*Gliomaps* visually resembled the original ^11^C-methionine positron emission tomography images. The residual error between *Gliomaps* and the original images from test datasets was 0.07 ± 0.04 (mean ± SD) in tumor-to-normal tissue ratio. The Sørensen-Dice coefficient between the lesions predicted by *Gliomap* and ^11^C-methionine positron emission tomography reached 0.88 ± 0.07 (mean ± SD) at a threshold of tumor-to-normal tissue ratio of 1.5. The absolute values of *Gliomap* showed a significant positive correlation with tumor cell density (*P *= .02).

**Conclusion:**

The present research demonstrates that the *Gliomap,* generated from contrast-enhanced magnetic resonance imaging using generative artificial intelligence, is a promising imaging surrogate for visualizing tumor cell density in newly diagnosed glioblastoma.

Key points

- ^11^C-methionine PET-like images named *“Gliomap”* can be generated from contrast-enhanced MRI
- *“Gliomap”* significantly correlated with tumor cell
- *“Gliomap”* is promising to visualize non-contrast-enhancing tumors in newly diagnosed glioblastoma

Importance of the study

Glioblastoma often features non-contrast-enhancing tumor (nCET) regions that are difficult to distinguish from vasogenic edema on standard MRI scans. Complete visualization of these nCET areas is crucial for effective surgical and radiation planning to improve patient outcomes. While ^11^C-methionine PET (MET-PET) can accurately map tumor cell density and identify nCET, its use is severely limited by high costs and lack of availability. This creates a critical gap in clinical practice, where clinicians need a more accessible tool to accurately delineate the full extent of a tumor. This study developed a conditional Generative Adversarial Network (cGAN) called *“Gliomap-GAN.”* This AI model learns to translate routinely acquired MRI scans into a MET-PET-like image, the *“Gliomap,”* which visualizes tumor cell density. With a lightweight model size of only 217.7 MB, the *Gliomap-GAN* can be easily integrated into existing hospital PACS servers, providing a practical, cost-effective solution to better define treatment targets and ultimately improve patient care.

Glioblastoma is classified as a CNS WHO Grade 4 tumor, distinguished by its aggressive proliferation and invasive characteristics. Typically, glioblastoma presents with non-contrast-enhancing lesions (nCET) adjacent to gadolinium-enhancing lesions.^1–3^ These nCET areas typically appear as high-intensity lesions on T2-weighted imaging (T2WI) and fluid-attenuated inversion recovery (FLAIR) sequences, while displaying low intensity on T1-weighted imaging (T1WI).^4–7^ A subtle disruption of the blood-brain barrier within nCET may be detectable through careful examination of subtraction images derived from gadolinium (Gd) enhanced and non-enhanced T1WI.^8^ Nonetheless, this observation alone does not provide a comprehensive understanding of tumor spread in the brain. Furthermore, vasogenic edema resulting from the space-occupying lesion presents similar imaging features, appearing as high-intensity on T2WI and low-intensity on T1WI, akin to nCET.^1^^,^^9–12^ Given the pivotal role of radical surgical resection alongside adequate chemotherapy and radiation therapy in treating glioblastoma patients, complete lesion visualization, including nCET, is essential.^2^^,^^3^^,^^13–18^

^11^C-methionine positron emission tomography (MET-PET) is neither yet regulatorily approved nor reimbursed by the health insurance system. However, it is widely used as a clinical research or non-insurance reimbursed agent for glioblastoma in Japan. MET-PET, which utilizes amino acid tracers, is one of the few imaging modalities capable of visualizing glioblastoma and can be considered a glioblastoma cell density map.^1^^,^^10^^,^^19–22^ This concept is further supported by a meta-analysis indicating that MET-PET exhibits the highest sensitivity and specificity in detecting tumor presence compared to other imaging techniques,^12^ accompanied by studies suggesting that MET-PET can effectively estimate tumor cell density.^1^^,^^19^^,^^20^ With the rapid advancement in artificial intelligence, the potential of a conditional Generative Adversarial Network (cGAN) is currently being extensively investigated in image synthesis.^23^ Based on the premise that minute signal changes corresponding to tumor cell presence can be identified by training a cGAN, a prior investigation indicated that a MET-PET-like image could be generated from Gd-enhanced T1WI (T1Gd).^24^ However, that investigation did not use other magnetic resonance imaging (MRI) sequences. This investigation leverages a unique glioblastoma cohort with MET-PET scans. It aims to expand on this concept by utilizing not only T1Gd but also T1WI and T2WI, with image normalization, before training the cGAN. The study aims to develop a cGAN (*Gliomap-GAN*) that can generate glioblastoma cell density maps (*Gliomap*) from contrast-enhanced MRI, utilizing MET-PET as a reference. Such technology is expected to aid clinicians in identifying potential nCET regions.

## Materials and methods

### Subjects and Materials

This study received approval from the Asahikawa Medical University Research Ethics Committee (approval number: 21041) and from the research ethics review boards of all participating institutions, confirming that all experiments will adhere to relevant guidelines and regulations in compliance with the Declaration of Helsinki. Written informed consent was waived for this research. Two cohorts were subjected to analysis. The first cohort was used to train and test the *Gliomap-GAN* model. It comprised 81 histologically confirmed glioblastoma patients who underwent both contrast-enhanced MRI and MET-PET. They were retrospectively collected from the following institutions: Osaka University Hospital (21 patients), Osaka International Cancer Institute (9 patients), Kagawa University Hospital (29 patients), and Hokuto Hospital (22 patients). Some of the cases were previously used in publications for different purposes.^1^^,^^11^ The histological diagnosis was based on the 2021 WHO classification of tumors of the central nervous system (CNS), 5th edition (WHO CNS 5). Only patients with tumors exhibiting contrast-enhancing lesions were included in this study, as the primary focus was to develop a technology for effectively visualizing tumor extension in typical glioblastoma-appearing lesions. The second cohort was used to externally validate the *Gliomap-GAN’s* performance in predicting tumor cell density by comparing the generated tumor cell density map and tissues obtained by image guidance. The tissues were obtained as part of routine clinical practice to confirm the extent of tumor spread at locations that are surgically accessible and safe. Sixteen tissue samples were obtained from five patients who underwent surgery at the Osaka International Cancer Institute and were not included in the first cohort. A portion of the data previously published was reused for this research.^11^

### ^11^C-methionine Positron Emission Tomography and Magnetic Resonance Imaging

MET-PET was performed at three different institutions. At Osaka University Hospital and Osaka International Cancer Institute, the PET scans were conducted using the Eminence-G system (Shimadzu Corporation, Kyoto, Japan). Each patient received an intravenous injection of ^11^C-methionine at a dose of 3 MBq/kg body weight. After 20 minutes post-injection, regional emission images were obtained for 12 minutes. At Kagawa University Hospital, PET scans were performed using the Biograph mCT64 PET/CT scanner (Siemens/CTI, Knoxville, TN, USA), with patients receiving intravenous ^11^C-methionine injections ranging from 182 to 448 MBq (mean dose: 316 ± 55.6 MBq). After 10 minutes of injection, regional emission images were captured for a period of 5 minutes. At Hokuto Hospital, PET scans were performed using the Discovery ST Elite (GE Healthcare Japan Co., Ltd, Tokyo, Japan), with patients receiving ^11^C-methionine in doses ranging from 375 to 459 MBq (mean dose: 416 ± 25 MBq). Regional emission images were obtained after 20 minutes of injection for 7 minutes.

As the cohort comprised multiple institutions, the imaging parameters for MRI varied among subjects, with images acquired from either 1.5 or 3.0 T scanners. However, all patients were required to have undergone T1-weighted imaging (T1WI), T2-weighted imaging (T2WI), and gadolinium-enhanced T1-weighted imaging (T1Gd) to be included in the cohort.

### Image Registration and Intensity Normalization for Magnetic Resonance Imaging

All MRIs, including T1WI, T2WI, and T1Gd, were registered, resliced, and normalized prior to being sent for downstream image processing. Initially, T1WI and T2WI were registered and resliced to T1Gd using the FMRIB Linear Image Registration Tool (FLIRT), followed by skull stripping using The Brain Extraction Tool (BET2), provided by the FMRIB Software Library (FSL).^25^^,^^26^ This process enables voxel-by-voxel analysis and extraction of signal intensity from the brain alone. A histogram of the signals with 256 bins was created, and the mode was identified. The signal intensity of the original image, prior to skull stripping, was divided by the mode multiplied by 75 to produce a normalized image as represented in the following equation (1), where *SI_Normalized_*, *SI_Original_*, and mode(*SI_256bins_*) stands for the normalized signal intensity, original signal intensity, and the mode of the signal intensity counted in 256 bins, respectively. This procedure was adopted to provide the best visual contrast in MRIs after intensity normalization. As a result, correlating the mode to the value of 75 within a dynamic range from 0 to 255 was chosen.


(1) $${SI}_{\mathrm{Normalized}}={SI}_{\mathrm{Original}}\times \frac{75}{{\mathrm{mode}(SI}_{256b\mathrm{ins}})}$$

Any value of the *SI*_Normalized_ exceeding 255 was ceilinged to 255. This procedure produced normalized T1WI (T1WI_Normalized_), T2WI (T2WI_Normalized_), and T1Gd (T1Gd_Normalized_).

### Z-Score Normalization of Contrast Enhancement

To fully utilize the information embedded in T1Gd, a subtraction image was created by subtracting T1Gd_Normalized_ from T1WI_Normalized_. T1Gd_Normalized_ and T1WI_Normalized_ were skull-stripped by BET2, followed by a voxel-by-voxel scatter plot analysis. We calculated the standard deviation of the residual from the estimated linear regression for each voxel and assigned this value to create a z-score image. Detailed information is provided in the supplementary material. This z-score image of contrast enhancement (zCE) is expected to enhance both the quantity and accuracy of the information provided by the unprocessed T1Gd. For further use of zCE downstream, zCE was multiplied by 51 with a maximum ceiling of 255. This procedure produced a normalized zCE with an 8-bit, 256-gradient (zCE_Normalized_), which allows it to be used as one of the RGB components, constrained by an 8-bit, 256-gradient.

### Image Registration and Intensity Normalization for ^11^C-Methionine Positron Emission Tomography

The MET-PET was normalized to the mean standard uptake value (SUV) of the cerebellum, which was determined by using the cerebellum region of the structural atlas from MNI152. Specifically, the MET-PET and MNI152 were registered and resliced to the T1Gd using FLIRT from FSL, which then allowed the registration of the cerebellum mask on MNI152 to T1Gd. The registered and resliced MET-PET images were normalized by dividing them by the mean SUV of the cerebellum. This process transformed the MET-PET data from SUV (MET-PET_SUV_) to a tumor-to-normal tissue ratio (MET-PET_T/N ratio_), reducing intersubject variability and enabling further analysis across the entire cohort. Furthermore, the MET-PET_T/N ratio_ was multiplied by 51 with a maximum ceiling of 255, so that the MET-PET_T/N ratio_ can be used as a target image for the pix2pix algorithm, which is constrained by an 8-bit, 256-gradient image-to-image comparison.

### Source and Target Image Preparation

The current research aims to construct a conditional cGAN (*Gliomap-GAN*) to generate a glioblastoma’s cell density map (*Gliomap*) from contrast-enhanced structural MRI. TIWI, T2WI, and zCE were combined into a single RGB image by assigning T1WI’s, T2WI’s, and zCE’s normalized 8-bit signal intensities in 256 gradients in red, green, and blue, respectively. The RGB obtained from MRI was used as the source image, while the MET-PET_Normalized_ was used as the target image. Sixty-five subjects were randomly selected to construct the training dataset. The remaining 16 subjects were assigned to construct the test dataset. The training dataset consisted of 2459 pairs of MRI and MET-PET images, which were then augmented to 4,918 pairs by mirroring the image horizontally. The test dataset consisted of 593 pairs.

### Conditional Generative Adversarial Network

The “pix2pix,” a well-described conditional generative adversarial network (cGAN), was used to construct a model that can generate MET-PET images from T1WI, T2WI, and zCE images. The architecture of pix2pix contains a generator with a U-Net-based architecture (*G*) and a discriminator represented by a convolutional PatchGAN classifier (*D*).^27–29^ The objective of cGAN is to minimize the loss function of cGAN composed of *G* and *D*, while *G* is trying to minimize it, and *D* is trying to maximize it. The original pix2pix code was obtained through “Pytorch” (https://github.com/junyanz/pytorch-CycleGAN-and-pix2pix, accessed on March 22, 2025),^28^^,^^30^ which was used without significant modification for this research. The network was trained for 200 epochs on a Windows 11 workstation equipped with an Intel Xeon Bronze 3204 1.90 GHz CPU, 192 GB of random-access memory, and an NVIDIA RTX A5500 GPU with 24 GB of graphics memory. The RGB color-coded MRI data from the training dataset were used as source data, and the corresponding MET-PET_T/N ratio_ was used as target data to train the network. Subsequently, the obtained U-Net-based architecture (*G*) was used to generate the *Gliomap*, which was compared pixel-by-pixel with the corresponding MET-PET_T/N ratio_. The overall accuracy was visually evaluated with a quantitative assessment by calculating the residual error.

### Image Reconstruction Quality Measurements

A quantitative assessment of the reconstructed *Gliomap* was performed by comparing it with the original MET-PET_T/N ratio_, measuring the differences in individual voxels’ T/N ratio, peak, and regular signal-to-noise ratio (SNR), as well as the structural similarity index (SSIM). Peak/regular SNR and SSIM were calculated by the corresponding function supplied by the Image Processing Toolbox of MATLAB R2025a (MathWorks, Natick, MA, USA). Furthermore, the Sørensen-Dice score was calculated between the lesions predicted by *Gliomap* and the MET-PET_T/N ratio_ as a function of a given *Gliomap* value or MET-PET_T/N ratio_ threshold. Finally, the ratio between the lesions expected from *Gliomap* or MET-PET_T/N ratio_ and T1Gd was calculated as a function of a given *Gliomap* value or MET-PET_T/N ratio_ threshold.

### Intraoperative Image-Guided Tissue Sampling

Tissue sampling was performed through biopsies at the earliest stage of operation to minimize the effects of brain shift. Visual confirmation of anatomical landmarks, such as the cortical veins and sulci, verified the accuracy of the navigation system.^1^^,^^11^^,^^19^ A total of 16 tissues were randomly sampled. The sampling locations were stored within the navigation system, using Digital Imaging and Communications in Medicine (DICOM) coordinates, following stereotactic tissue sampling of the thin-slice T1Gd. These DICOM coordinates were then converted into the corresponding DICOM coordinates of the *Gliomap*. Average and standard deviation within a 1-cm^3^ voxel-of-interest at the target were obtained using in-house software developed in MATLAB R2025a (MathWorks, Natick, MA, USA). Formalin-fixed specimens were embedded in paraffin for histopathological examination. Hematoxylin and eosin-stained specimens were evaluated to calculate the tumor cell density. Cell counting was performed at ×400 magnification under light microscopy (Nikon, Tokyo, Japan).^11^

## Results

### Effectiveness of the Image Normalization Procedure

**Figure 1.**
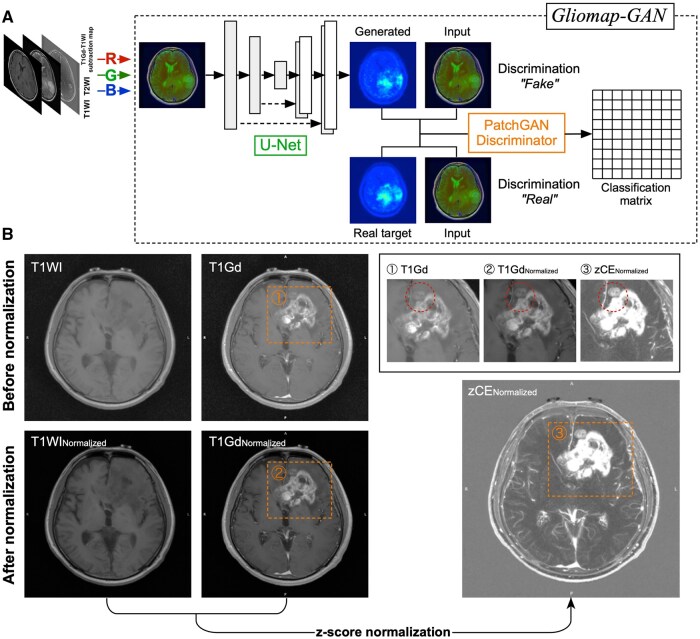
The pix2pix cGAN named *“Gliomap-GAN”* was trained using an RGB color-coded MRI derived from a normalized T1WI, T2WI, and zCE. The target image was the corresponding normalized MET-PET. Further details of the network can be found elsewhere.^27^^,^^28^ (A) The entire procedure for creating zCE_Normalized_ from T1WI and T1Gd is presented. Both images were first normalized through the process described in Figure 1, followed by a z-score-based subtraction. The dynamic range of the subtraction image was further expanded from 0 to 255. A subtle contrast enhancement that is difficult to identify in the original image (1) can be identified in zCE_Normalized_ (3), highlighted with a red dotted circle. (B).

Figure 1A illustrates the overall workflow and the effect of image intensity normalization as a preprocessing step for training the *Gliomap-GAN*. The change in the distribution of signal intensities of different MR sequences before and after applying the image normalization procedure is shown in (Figure S1). While the original T1WI, T2WI, and T1Gd images presented a wide range of variability in signal intensities, the normalization procedure reduced this variability. Furthermore, the variable data range was normalized to 256 levels, which is expected to facilitate the learning process of the downstream cGAN. Figure 1B demonstrates the effect of z-score normalization of contrast enhancement. zCE allows for the identification of even the slightest contrast enhancement that is difficult to identify on conventional T1Gd. Furthermore, normalizing the z-score to 256 levels (zCE: zCE_Normalized_) enabled equalizing the data range, which varies among subjects.

### “Gliomap-GAN” Model Training Process

The objective of training the *Gliomap-GAN* is to minimize the loss function of cGAN, composed of a generator (*G*) and a discriminator (*D*), while *G* tries to minimize it, and *D* behaves in the opposite direction. Thus, successful training is characterized by an increase in the loss of *G* and a decrease in the loss of *D, ultimately* stabilizing at a point where learning is saturated (Figure 1A). The loss function of the generator (*G*) gradually increased, accompanied by a corresponding decrease in the loss function of the discriminator (*D*), which stabilized around the 100th epoch (Figure S2A and S2B). Overall, both the generator and the discriminator underwent a stable training process. It is known that the L1 loss is not a reliable indicator for evaluating the training quality of cGAN (Figure S2C). The difference in each pixel’s value between the MET-PET_Normalized_ and the generated *Gliomap*, which is calculated as the residual, remained relatively constant during the training process (Figure S2D).

### Generation of “Gliomap” from MRI on a Test Dataset

**Figure 2.**
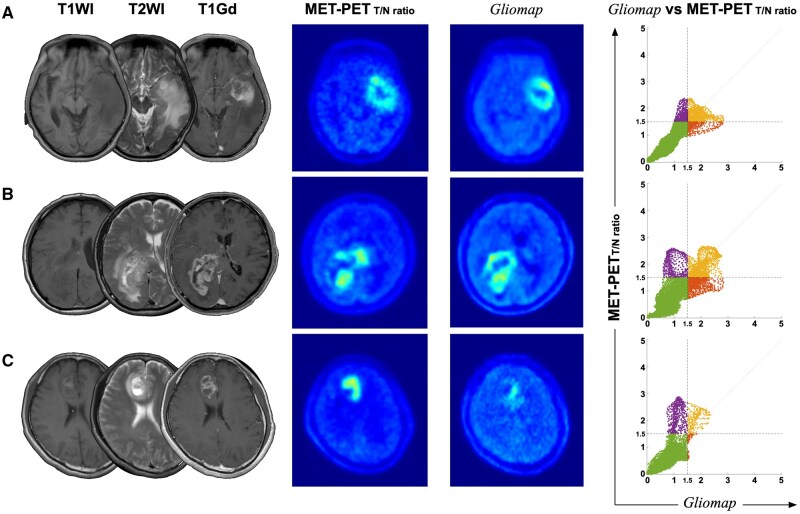
Several typical examples comparing the original MET-PETT/N ratio and the “Gliomap” images are presented. Example (A) shows a generation of “Gliomap” from MRI, perfectly resembling the MET-PETT/N ratio. The scatter plot comparing the generated and ground truth images showed an excellent correlation. Example (B) shows a case where some regions were overestimated in the “Gliomap.” Example (C) shows a case where a significant underestimation was observed in the “Gliomap.”

**Figure 3.**
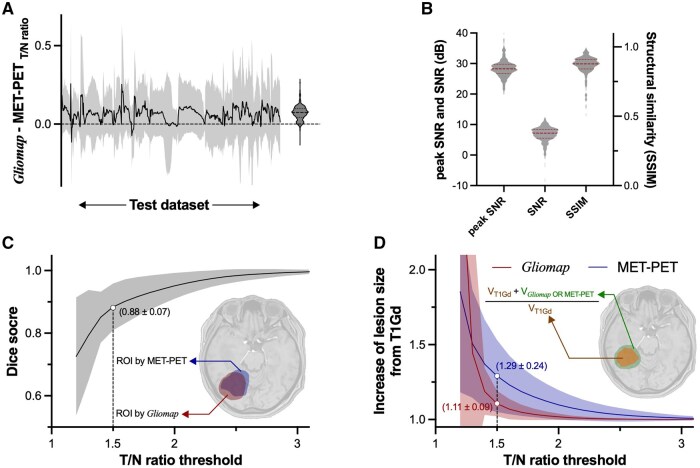
The residual error between the *“Gliomap”* and MET-PET_T/N ratio_ of all 593 test datasets, including those facing failed reconstruction, was 0.07 ± 0.04 (mean ± SD) with a tendency to overestimate methionine uptake. (A) Three image quality metrics are presented. (B) The Sørensen-Dice coefficient between the lesions predicted by *“Gliomap”* and the MET-PET_T/N ratio_ increased as the threshold increased, reaching 0.88 ± 0.07 (mean ± SD) at an MET-PET_T/N ratio_ of 1.5. (C) The volume expansion ratios were 1.29 ± 0.24 for MET-PET_T/N ratio_ and 1.11 ± 0.09 for *“Gliomap”*, respectively, at a threshold of 1.5 (mean ± SD). V_T1Gd_: Volume of gadolinium-enhancing lesion on T1Gd, V_*Gliomap*_: Volume of lesion predicted by *“Gliomap”* at a given threshold, V_MET-PET_: Volume of lesion predicted by *“Gliomap”* at a given threshold. (D).

Once the training dataset was built, the generator (*G*) of *Gliomap-GAN* was used to generate the *Gliomap* from MRI on the reserved test dataset. Several representative cases are shown in Figure 2, and the image generation user interface is presented in Supplementary Video 1. Overall, the *Gliomap* resembled the original image (MET-PET_Normalized_). However, careful observation revealed cases where the ­*Gliomap* exhibited a more pronounced lesion than MET-PET_Normalized_ (Figure 2B) and vice versa (Figure 2C). Furthermore, a rare case was identified where the generation of the image itself failed during reconstruction (Figure S3). One can quantitatively evaluate the generated image’s accuracy by plotting the pixel value of the *Gliomap* as a function of MET-PET_Normalized_ pixel-wise. The residual error between the *Gliomap* and MET-PET_Normalized_ of all 593 test datasets, including those with failed reconstruction, was 0.07 ± 0.04 (mean ± SD) in the tumor-to-normal tissue ratio (Figure 3A). The mean ± SD of the peak and regular SNR of *Gliomap* were 28.5 ± 3.2 and 6.5 ± 3.8, respectively, where a peak SNR greater than 30 in an 8-bit image is considered high-quality (Figure 3B).^31^ On the other hand, the mean ± SD of SSIM was 0.86 ± 0.07 ­(Figure 3A). An SSIM of greater than 0.9 is considered high-quality.^32^  Figure 3C shows the Sørensen-Dice score between the lesions predicted by *Gliomap* and the MET-PET_T/N ratio_ as a function of a given MET-PET_T/N ratio_ threshold. The Sørensen-Dice coefficient increased as the threshold increased, reaching 0.88 ± 0.07 (mean ± SD) at an MET-PET_T/N ratio_ of 1.5. Figure 3D shows the increase in lesion size from T1Gd by adding information from *Gliomap* or MET-PET_T/N ratio_ as a function of a given MET-PET_T/N ratio_ threshold. Both images identified possible nCET outside of gadolinium-enhancing lesions visualized on T1Gd. The volume expansion ratios were 1.29 ± 0.24 for MET-PET_T/N ratio_ and 1.11 ± 0.09 for *Gliomap*, respectively, at an MET-PET_T/N ratio_ of 1.5 (mean ± SD).

### Validation of “Gliomap” by Intraoperative Image-Guided Tissue Sampling

**Figure 4.**
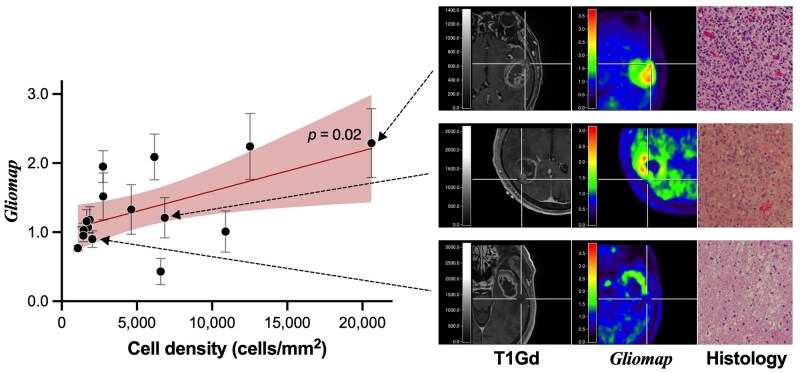
The absolute value of the *“Gliomap”* and the tumor cell density obtained from image-guided tissue sampling showed a significant positive correlation (*P *= .02).

Finally, the *“Gliomap”* was validated by tissues sampled under image guidance during surgery. The *“Gliomap”* absolute value is designed to correspond to the MET-PET_T/N ratio_. The absolute value of the *“Gliomap”* and the tumor cell density showed a significant positive correlation (*P *= .02, Figure 4). The linear regression model was expressed as follows.


(2) $$\mathrm{Gliomap}=5.8\;\times \;10^{-5}\times [\mathrm{Cell}\;\mathrm{density}\;(\mathrm{cells}/{mm}^{2})]+1.0$$

## Discussion

Previous studies have shown that significant tumor cells reside within nCET, necessitating the incorporation of this lesion into surgical or radiation oncological treatment targets. The recent movement to extend the planned surgical resection target into the high-intensity area on the T2-weighted image fluid-attenuated inversion recovery (T2-FLAIR), also known as the “FLAIRectomy,” is based on these observations.^2^^,^^3^^,^^6^^,^^14–18^^,^^33–38^ On the other hand, a significant challenge is posed when identifying nCET, which accompanies glioblastoma. The high-intensity lesion on T2-FLAIR represents either vasogenic edema resulting from the brain’s reaction to the lesion or true nCET.^6^ While PET using amino acid tracers is reported to be one of the most reliable imaging modalities for detecting nCET, its availability is limited due to economic, social, and logistical issues.^12^^,^^39^ Thus, MRI, one of the most easily accessible image modalities, is desired to exhibit the capability to visualize nCET. Recent advancements in artificial intelligence (AI) have given rise to a technology called Generative AI. Generative AIs are sets of artificial intelligence models that could generate new content, such as images, audio, and text, by learning existing data. In medical images, Generative AI is expected to deliver a technology that can not only shorten image acquisition time but also create synthetic images that are easier to comprehend. The current research aimed to build a Generative AI model that can generate an amino acid PET-like image named *“Gliomap”* from MR images acquired in routine daily practice. If successful, the developed technology is expected to enable the identification of nCET using conventional MR images alone.

cGAN is situated among various Generative AI models that focus on generating new images from existing data. The network’s character is unique in that it consists of two algorithms that counteract each other to augment the accuracy of the generated image while minimizing overfitting. The current study employed “pix2pix,” one of the most well-studied conditional generative adversarial networks (cGANs) in image generation (Figure 1).^27–29^ The first algorithm, the generator, is a U-Net-based convolutional neural network trained to “recreate” the target image from the source image. The second algorithm, the discriminator, will function in a way that prevents the first algorithm from overfitting. In the current study, three layers of data were used as input: T1-weighted image (T1WI), T2-weighted image (T2WI), and a z-score normalized image of the Gd-enhanced T1WI (T1Gd) minus the T1WI. We did not directly use T1Gd as one of the data sources, considering that T1WI already entails significant data, especially on non-enhancing tissues.

Instead, the key contribution of T1Gd lies in the information regarding the “different magnitudes of contrast enhancement” in each voxel. Hence, a voxel-wise subtraction-like image was considered more preferred in the current scenario. The method for image normalization was handled with care in this research. Images should be normalized without observing the characteristics of the entire cohort (Figure 1 and Figure S1). For example, normalizing images based on the distribution of signal intensities across the entire cohort may not be ideal, particularly in situations where each input dataset must be normalized prospectively. In the present research, all images were normalized *a priori* by fixing the dynamic range from 0 to 255, aiming for the mode of the distributed data to be 75. The proposed approach enables image normalization without requiring observation of other data from the entire cohort.

Furthermore, taking advantage of the original pix2pix’s ability to handle three layers of information as input data, the T1WI, T2WI, and z-score image of contrast enhancement (zCE) were combined into a single RGB image (Figure 1). A previous study that attempted to solve the same problem used only T1Gd as input.^24^ Qualitative information retrieved not only from T1Gd but also from T2WI or FLAIR is crucial to identify nCET. Thus, efficiently utilizing all available images is key to generating accurate images that aid in nCET visualization. On the other hand, the current investigation chose to use FLAIR rather than T2WI, as image characteristics of FLAIR could easily be affected by the “inversion time” parameter that is preferably used at individual institutions. The residual error between the MET-PET_T/N ratio_ and the *“Gliomap”* of the test dataset was as low as 0.07 ± 0.04, with moderate image quality deterioration of *Gliomap* compared to the MET-PET_T/N ratio_ ­(Figure 3A and B). Although a direct comparison cannot be made between the present and previous research, the proposed method at least appears to be as effective as the last report.^24^ Furthermore, the geometric visualization of the possible nCET showed similarity between the two image modalities, as evaluated by the Sørensen-Dice score and lesion-size expansion ratio from gadolinium-enhancing lesions (Figure 3C and D).

Finally, the *“Gliomap”* showed a significant correlation with tumor cell density (Figure 4). The derived slope of the linear regression equation was 5.8 × 10^−5^, which is one-third of that derived from the MET-PET_T/N ratio_ in our previous study.^1^ It is worth noting that the image normalization methods employed in the current and prior studies differ, and further confirmation is deemed necessary.

While the proposed method appears to offer a promising solution for intuitively and effectively identifying glioblastoma’s nCET, several critical issues need to be addressed. First, there were cases where image reconstruction failed, generating a corrupted image that was useless in practice (Figure S2). The developed generator may not have functioned as expected under certain conditions, necessitating a more thorough investigation to identify the cause. Increasing the sample size for training the model can help overcome the model’s overfitting to specific situations, thereby improving the generator’s performance. Secondly, there was a tendency for the generated image to overestimate methionine uptake (Figure 3A). This tendency was particularly evident in areas with low methionine uptake (Figure 3D). It is possible that the trained model was affected by the high-intensity signal deriving from T2WI, expanding the presumed methionine uptake area. Finally, the correlation between *Gliomap* and 5-aminolevulinic acid (ALA) fluorescence was not investigated. In fact, RANO resect, RANO recurrent glioblastoma, RANO radiotherapy, and RANO/PET have recently published a consensus statement that encourages the incorporation of 5-ALA fluorescence in identifying glioblastoma’s nCET.^40^ The value of *Gliomap* must be critically evaluated in real case scenarios.

*Gliomap-GAN* is a lightweight model with a size of 217.7 MB; thus, it should be easily incorporated into a picture archiving and communication system (PACS) server for background image reconstruction. Its most ideal integration into daily clinical practice is to automatically reconstruct and display *Gliomap* alongside other MRI sequences on the DICOM viewer.

## Conclusion

The present research presented that generating MET-PET-like images named *“Gliomap”* from contrast-enhanced MRI, along with T1WI and T2WI, is possible using generative AI and appropriate image normalization procedures. Although further refinement is necessary, the *“Gliomap”* generated from routinely available clinical MRI could be a practical solution for estimating glioblastoma’s cell density and further identifying nCET without the need for sophisticated resources such as amino acid PET.

## Supplementary Material

vdaf227_Supplementary_Data

## Data Availability

Custom code and raw data are available upon request.
