# Supplementary material for: Gliomap-GAN: A conditional generative adversarial network to visualize glioblastoma’s cell density from contrast-enhanced magnetic resonance imaging
Source: Neurooncol Adv. 2025 Oct 21;8(1):vdaf227. doi: 10.1093/noajnl/vdaf227 (PMC13010284; doi:10.1093/noajnl/vdaf227)
Supplement: vdaf227_Supplementary_Data [file vdaf227_supplementary_data.zip › Supplementary materials (Revised - clean).docx]

***Gliomap-GAN*: A conditional Generative Adversarial Network to visualize glioblastoma’s cell density from** **contrast-enhanced magnetic resonance imaging**

Manabu Kinoshita^1^, Keisuke Miyake^2^, Wataru Ide^3^, Hideyuki Arita^4^, Kayako Isohashi^5^, Jun Hatazawa^6^, and Haruhiko Kishima^7^

^1^Department of Neurosurgery, Asahikawa Medical University, Asahikawa, Japan

^2^Department of Neurosurgery, Kagawa University Faculty of Medicine / Graduate School of Medicine, Kita District, Japan

^3^Department of Neurosurgery, Hokuto Hospital, Obihiro, Japan

^4^Department of Neurosurgery, Osaka International Cancer Institute, Osaka, Japan

^5^Department of Radiology, Osaka University, Graduate School of Medicine, Suita, Japan

^6^Department of Physics, Osaka University, Graduate School of Science, Suita, Japan

^7^Department of Neurosurgery, Osaka University, Graduate School of Medicine, Suita, Japan

**Corresponding author:** Manabu Kinoshita M.D., Ph.D.

Department of Neurosurgery, Asahikawa Medical University

Midorigaoka-higashi 2-1-1-1, Asahikawa, Hokkaido 078-8510 Japan

Email: mail@manabukinoshita.com

TEL: +81-166-68-2594, FAX: +81-166-68-2599

*Z-score normalization of contrast enhancement*

To fully utilize the information embedded in T1Gd, a subtraction image was created by subtracting T1Gd_Normalized_ from T1WI_Normalized_. T1Gd_Normalized_ and T1WI_Normalized_ were skull-stripped by BET2, followed by a voxel-by-voxel scatter plot analysis. We calculated the standard deviation of the residual from the estimated linear regression for each voxel and assigned this value to create a z-score image.

A Linear regression fitting was applied to voxel values obtained by T1Gd_Normalized_ and T1WI_Normalized_, which can be expressed as follows:

$${T1Gd}_{Normalized}= \alpha\times{T1WI}_{Normalized}+ \beta$$

By solving *α* and *β*, one can now determine the linear correlation of T1Gd_Normalized_ and T1WI_Normalized_ of the entire brain. Next, the magnitude of residual from this solved linear regression line for any particular voxel (*i*) can be expressed as follows:

$${residual}_{i}= \frac{{{(T1Gd}_{Normalized})}_{i}-\alpha\times\left( {T1WI}_{Normalized} \right)_{i}-\beta}{\sqrt{\alpha^{2}+1}}$$

Finally, the z-score image of contrast enhancement (zCE) of each data point was defined as follows:

$$zCE= \frac{{residual}_{i}-\mu}{\rho}$$

where μ and σ are the means and SD of “residual*_i_*”, respectively.

This z-score image of contrast enhancement (zCE) is expected to enhance both the quantity and accuracy of the information provided by the unprocessed T1Gd. For further use of zCE downstream, zCE was multiplied by 51 with a maximum ceiling of 255. This procedure produced a normalized zCE with an 8-bit, 256-gradient (zCE_Normalized_), which allows it to be used as one of the RGB components, constrained by an 8-bit, 256-gradient.

**Supplementary Figure 1**


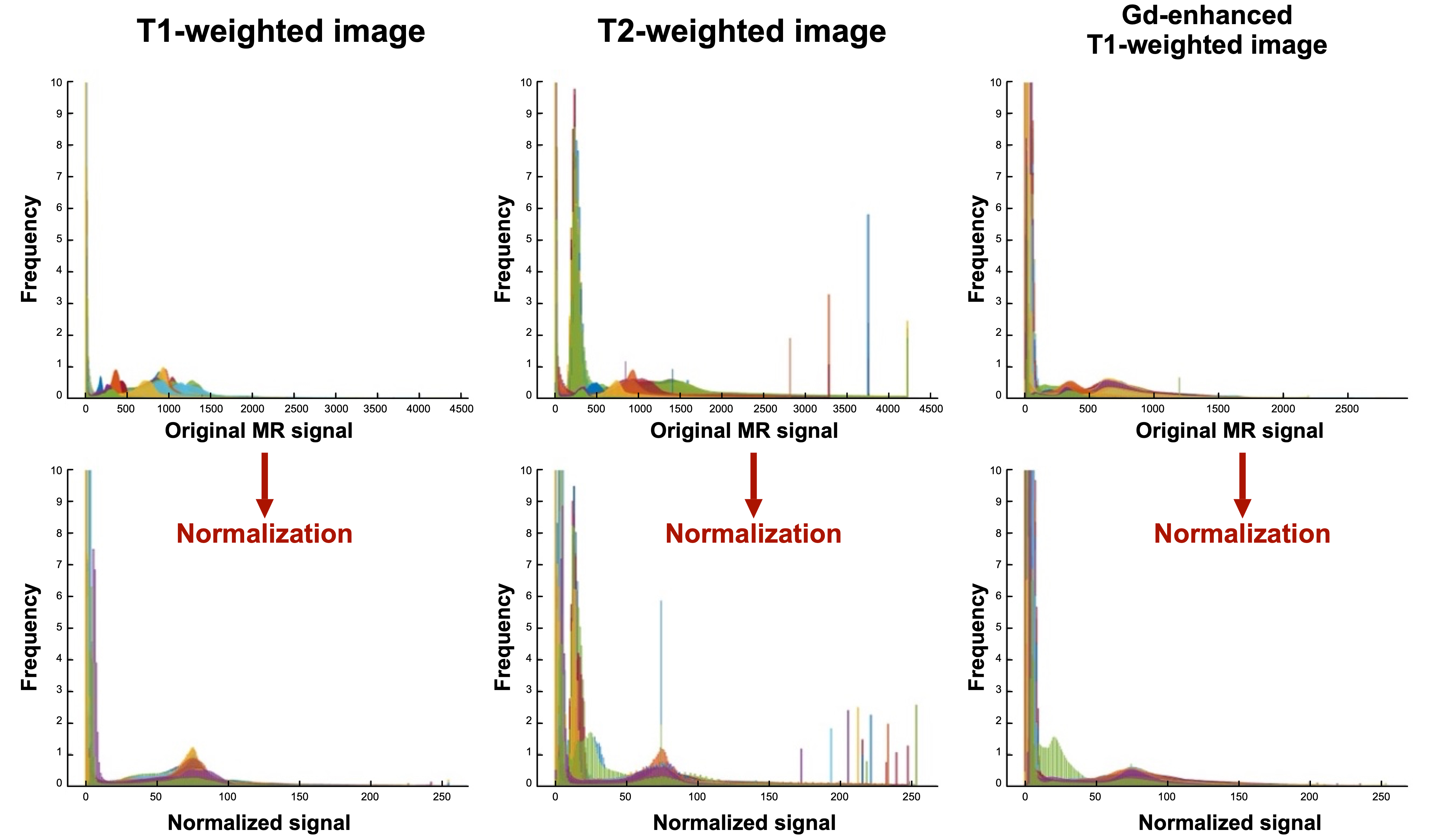


Outcomes of the intensity normalization procedure are presented. Each set of images was normalized, aiming the mode of the signal intensity distribution to meet a value of 75 with a dynamic range from 0 to 255. The second peaks of the histogram of the signal intensities are centered around 75 after going through this process.

**Supplementary Figure 2**


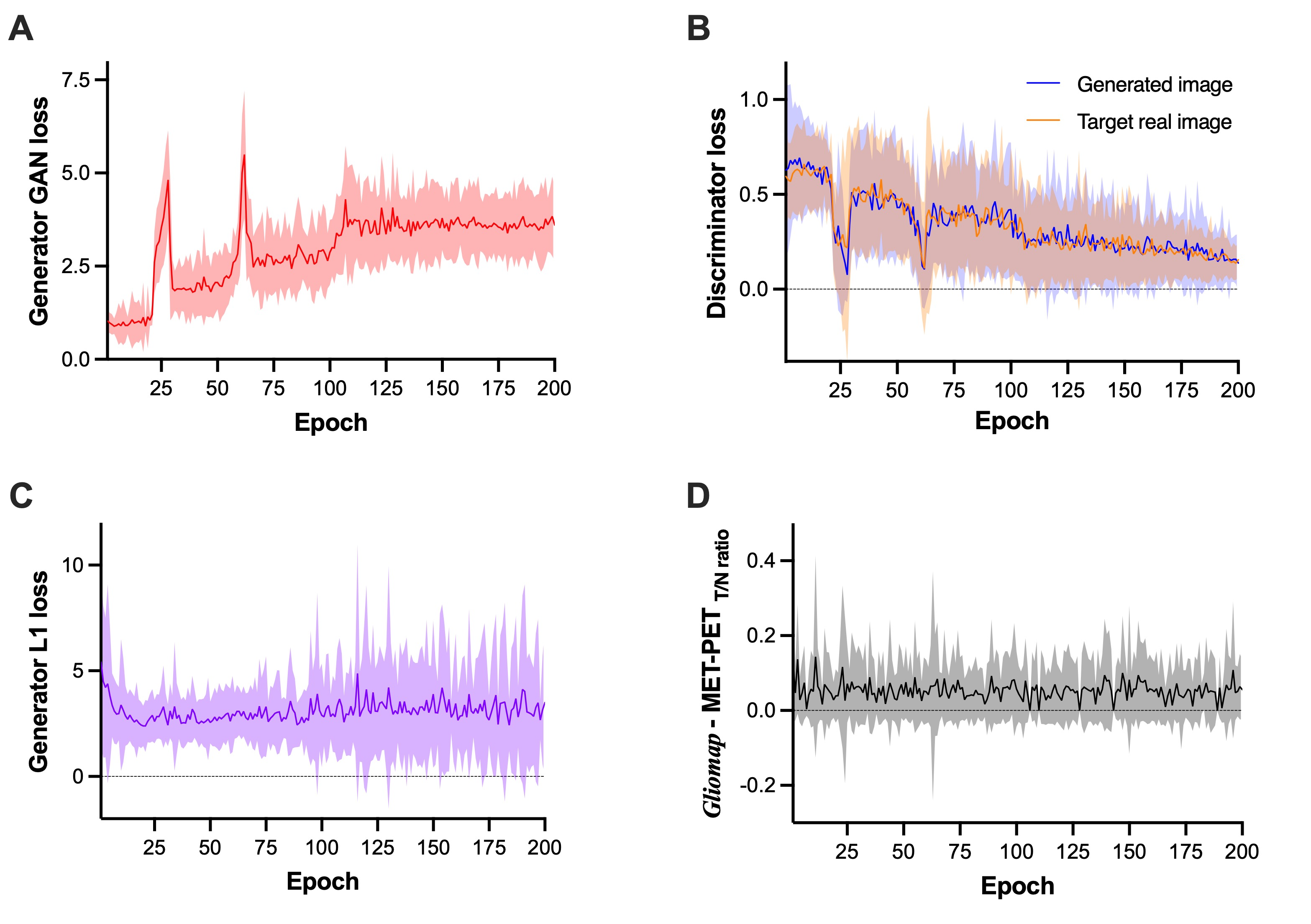


The training process of the network is presented. The objective of training a cGAN is to minimize the loss function of a cGAN composed of a generator and a discriminator, while the generator tries to minimize it, and the discriminator behaves in the opposite direction. Thus, successful training is represented by an increase in the loss of the generator and a decrease in the discriminator’s loss, stabilizing at a point where the learning is saturated. The loss function of the generator gradually increased (A), accompanied by a gradual decrease of the discriminator’s loss function (B), stabilizing at around the 100th epoch. L1 loss is not a reliable indicator for evaluating the training quality of cGAN (C). The difference in each pixel’s value between the Target real image and the Generated image, which is calculated as the residual, did not significantly change during the training process (D).

**Supplementary Figure 3**

**
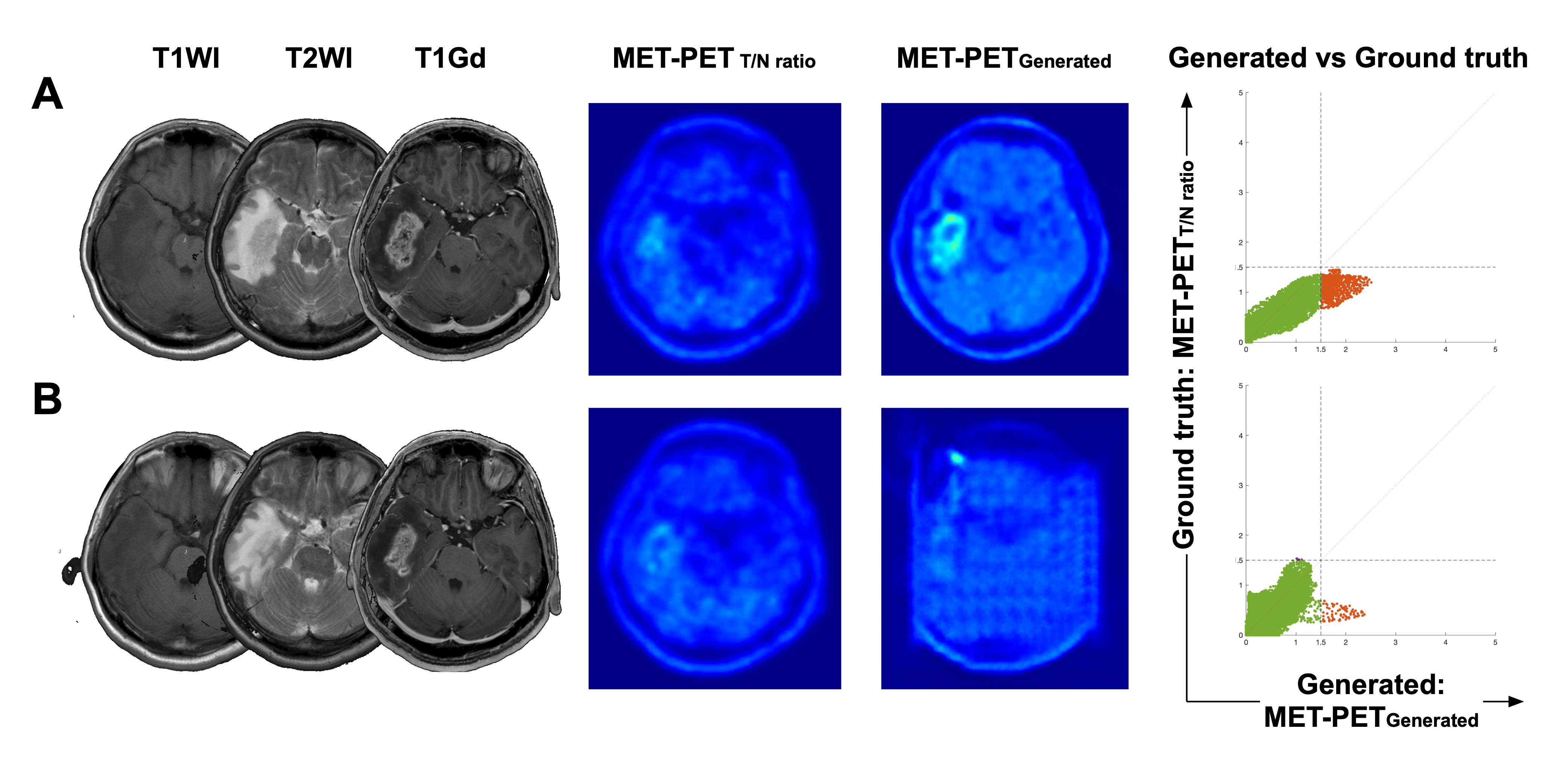
**

A case of failure in generating *“Gliomap”* is presented. While the images on panel (B) were derived from a slice adjacent to the images on panel (A), the image quality of the generated *“Gliomap”* was far inferior to expectation.
